# Silicon carbide, the next-generation integrated platform for quantum technology

**DOI:** 10.1038/s41377-024-01515-0

**Published:** 2024-08-29

**Authors:** Haiyan Ou

**Affiliations:** Department of Electrical and Photonics Engineering, Technical University of Denmark, Kgs. Lyngby, 2800 Denmark

**Keywords:** Other photonics, Nonlinear optics

## Abstract

Silicon carbide (SiC) is emerging as a promising material platform for quantum photonic integrated circuits (QPICs). A quantum light source is one of the fundamental building blocks for QPICs. A high-performance quantum light source from SiC platform will facilitate SiC’s infiltration into QPICs.

Nowadays, we are in the midst of the so-called second quantum technology revolution, which aims to bring paradigm-shifting solutions to critically important applications in every corner of human life. Examples include secure communication that detects any form of spying, efficient computation that is exponentially faster than any classical version, and quantum sensing that exploits the unique quantum properties to provide unprecedented sensitivity. To tackle these grand challenges, we need to develop groundbreaking technology covering all levels, from materials through devices to systems.

Quantum photonic integrated circuits (QPICs) are the technology of choice for various applications because of their compactness, energy efficiency, low cost, high performance and potential for up-scalability^1^. The basic building blocks for QPICs include quantum light sources, passive and active devices for light manipulation such as beam splitters, filters, wavelength conversion, modulation, etc., and quantum detectors. Despite limited commercial availability, silicon carbide on insulator (SiCOI) is emerging as the next-generation material platform for quantum technology^2^. As seen in Table 1, in comparison to the existing material platforms of silicon on insulator (SOI), silicon nitride (SiN) and lithium niobate on insulator (LNOI)^3^, SiCOI shows advantages in the following aspects: broad transparency with < 1 dB/cm waveguide loss demonstrated from ~ 400 nm to ~ 5000 nm; electro-optic, and thermal switching/modulation/tuning mechanisms demonstrated; second harmonic and other nonlinear light generation and color center-based single photon sources demonstrated. Some reported color centers in SiC work at telecommunication wavelength ranges and room temperature, and have long spin coherence times up to 5 s^4^, which holds promising potential applications in quantum communications, quantum sensing, and quantum computing.

**Table 1:** Comparison of SiCOI with the major material platforms of SOI, SiN and LNOI

Property	SOI	SiN	LNOI	SiCOI
Maturity	Very mature	Mature	Limited commercial availability	Very limited commercial availability
Transparency window	From 1100 nm to 3700 nm^a^^9^	Very low loss from 400 nm to 7500 nm^10^	Low loss from 350 nm to 5000 nm^11^	Low loss from 387 nm to 5600 nm^b^^12^
Modulation mechanisms	Thermal, carrier, and MEMS based	Thermal	Electro-otpical, piezoelectrical and thermal	Electro-optical and thermal
Nonlinearity	Third-order nonlinearity, but strong nonlinear absorption at telecom wavelength	Ultrahigh-Q resonators for third-order nonlinear light generation	Second harmonic and other nonlinear light generation	Both second-order and third-order nonlinearity for wavelength conversion and nonlinear light generation
Mechanism for quantum-state generation	SFWM	SFWM	SPDC	SFWM, Color center- based single photon source

^a^the onset of mid-infrared absorption in silica

^b^for 4H-SiC

Among the basic building blocks, the quantum light source is pivotal. Depending on the applications, quantum light sources are versatile. One type is called single photon source, where a single photon is emitted from a single defect or atom, such as color centers and quantum dots. The other type is generated through the nonlinearity of the material, known as spontaneous parametric down-conversion (SPDC) and spontaneous four-wave mixing (SFWM), corresponding to second-order (χ^2^) and third-order (χ^3^) nonlinearity respectively^5^. SiC has the unique advantages of possessing both second-order and third-order nonlinearity, along with versatile color centers. The corresponding quantum light sources are schematically shown in Fig. 1.

**Fig. 1:**
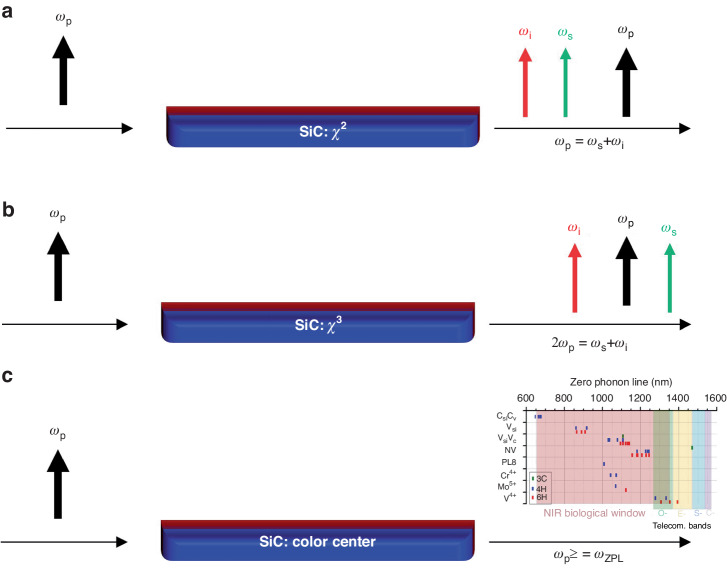
Schematic of three types of quantum light sources based on the SiCOI platform. **a** spontaneous parametric down-conversion (SPDC), **b** spontaneous four-wave mixing (SFWM) and **c** color centers. p pump, i idler, s signal, ZPL zero phonon line, ω angular frequency

As an example of a quantum light source generated from nonlinearity, in a newly published paper in *Light: Science & Applications* by Anouar Rahmouni, Xiao Tang, Thomas Gerrits, Oliver Slattery and Lijun Ma from the National Institute of Standards and Technology, USA, and Ruixuan Wang, Jinwei Li and Qing Li from the Department of Electrical and Computer Engineering, Carnegie Mellon University, USA, proposes entangled photon pair generation based on third-order nonlinearity, i.e., four-wave mixing (FWM), in the integrated SiC platform^6^. For the first time, they have generated strongly correlated photon pairs at the telecom C-band wavelength by implementing spontaneous four-wave mixing in a compact microring resonator in the 4H SiCOI platform. The entangled source has been measured to have a maximum coincidence-to-accidental ratio greater than 600 for an on-chip photon pair rate of (9 ± 1) × 10^3^ pairs/s and a pump power of 0.17 mW, a heralded *g*^(2)^ (0) on the order of 10^−3^, and a visibility of two-photon interference fringe exceeding 99%. These results are comparable to those obtained from more mature nonlinear integrated platforms such as silicon, silicon nitride and aluminum gallium arsenide.

The above observation for the first time demonstrated entangled photon pair generation from an integrated 4H-SiCOI platform. Quantum information systems are very sensitive to noise, which can greatly reduce the fidelity of quantum information. This work achieved a high CAR (coincidence-to-accidental ratio) of photon pair generation (>600) and a visibility of two-photon interference fringe (>99%), experimentally demonstrating that the noise level in such an SiC platform satisfies the requirements of quantum information devices based on single photons.

Looking forward, the performance of entangled photon pair generation from spontaneous four-wave mixing of 4H-SiCOI is expected to improve as the 4H-SiC material and nanofabrication technology develop, broadening its quantum applications^7^. 4H-SiC possesses both second-order and third-order nonlinearity. Quantum light sources based on second-order nonlinearities such as second harmonic generation and sum frequency generation, are expected in the near future, which will also provide flexibility for wavelength conversion.

It is foreseeable that the potential of the SiCOI platform will be gradually released as material quality and nanofabrication technology advance. Among the widely available polytype of SiC, 3 C and 4H, 4H-SiC shows the best crystal quality benefitting from its extensive application in power electronics. Therefore the above observation is from this polytype. 3C-SiC possesses slightly different optical properties. For now, 3C-SiCOI has demonstrated better performance in electro-optical modulation than 4H-SiCOI^8^. This versatility incites competition among different polytypes and speeds up the technology maturity, which in general helps SiC emerge as a promising material platform for QPIC.

## References

[1] Wang, J. W. et al. Integrated photonic quantum technologies. *Nat. Photonics* **14**, 273–284 (2020). 10.1038/s41566-019-0532-1

[2] Guidry, M. A. et al. Quantum optics of soliton microcombs. *Nat. Photonics* **16**, 52–58 (2022). 10.1038/s41566-021-00901-z

[3] Saravi, S., Pertsch, T. & Setzpfandt, F. Lithium niobate on insulator: an emerging platform for integrated quantum photonics. *Adv. Optical Mater.* **9**, 2100789 (2021). 10.1002/adom.202100789

[4] Castelletto, S. et al. Silicon carbide photonics bridging quantum technology. *ACS Photonics* **9**, 1434–1457 (2022). 10.1021/acsphotonics.1c01775

[5] Caspani, L. et al. Integrated sources of photon quantum states based on nonlinear optics. *Light Sci. Appl.* **6**, e17100 (2017). 30167217 10.1038/lsa.2017.100 PMC6062040

[6] Rahmouni, A. et al. Entangled photon pair generation in an integrated SiC platform. *Light Sci. Appl.* **13**, 110 (2024). 38724516 10.1038/s41377-024-01443-z PMC11082171

[7] Zhang, Z. D. et al. Entangled photons enabled time-frequency-resolved coherent Raman spectroscopy and applications to electronic coherences at femtosecond scale. *Light Sci. Appl.* **11**, 274 (2022). 36104344 10.1038/s41377-022-00953-y PMC9474554

[8] Powell, K. et al. Integrated silicon carbide electro-optic modulator. *Nat. Commun.* **13**, 1851 (2022). 35383188 10.1038/s41467-022-29448-5 PMC8983721

[9] Chang, L. et al. CSOI: beyond silicon-on-insulator photonics. *Opt. Photonics N.* **33**, 24–31 (2022). 10.1364/OPN.33.1.000024

[10] Krückel, C. J. et al. Optical bandgap engineering in nonlinear silicon nitride waveguides. *Opt. Express* **25**, 15370–15380 (2017). 28788964 10.1364/OE.25.015370

[11] Zhu, D. et al. Integrated photonics on thin-film lithium niobate. *Adv. Opt. Photonics* **13**, 242–352 (2021). 10.1364/AOP.411024

[12] Afridi, A. A. et al. 4H–SiC microring resonators—Opportunities for nonlinear integrated optics. *Appl. Phys. Lett.* **124**, 170502 (2024). 10.1063/5.0198517

